# An Efficient Method for Genomic DNA Extraction from Different Molluscs Species

**DOI:** 10.3390/ijms12118086

**Published:** 2011-11-17

**Authors:** Jorge C. Pereira, Raquel Chaves, Estela Bastos, Alexandra Leitão, Henrique Guedes-Pinto

**Affiliations:** 1 Institute for Biotechnology and Bioengineering, Center of Genomics and Biotechnology, University of Trás-os-Montes and Alto Douro (IBB/CGB-UTAD), Apdo1013, Vila Real 5001-801, Portugal; E-Mails: jorgecpereira@portugalmail.pt (J.C.P.); ebastos@utad.pt (E.B.); h.gp@hotmail.com (H.G.-P.); 2 Department of Genetics and Biotechnology, University of Trás-os-Montes and Alto Douro, Apdo1013, Vila Real 5001-801, Portugal; 3 National Institute of Biological Resources (INRB, I.P.)/IPIMAR, Avenue 5 de Outubro s/n, Olhão P-8700-305, Portugal; E-Mail: aleitao@ipimar.pt

**Keywords:** molluscs, DNA, QuickGene-810, DNA yield, concentration and purity

## Abstract

The selection of a DNA extraction method is a critical step when subsequent analysis depends on the DNA quality and quantity. Unlike mammals, for which several capable DNA extraction methods have been developed, for molluscs the availability of optimized genomic DNA extraction protocols is clearly insufficient. Several aspects such as animal physiology, the type (e.g., adductor muscle or gills) or quantity of tissue, can explain the lack of efficiency (quality and yield) in molluscs genomic DNA extraction procedure. In an attempt to overcome these aspects, this work describes an efficient method for molluscs genomic DNA extraction that was tested in several species from different orders: Veneridae, Ostreidae, Anomiidae, Cardiidae (Bivalvia) and Muricidae (Gastropoda), with different weight sample tissues. The isolated DNA was of high molecular weight with high yield and purity, even with reduced quantities of tissue. Moreover, the genomic DNA isolated, demonstrated to be suitable for several downstream molecular techniques, such as PCR sequencing among others.

## 1. Introduction

Reliability, feasibility and reproducibility of molecular genetics studies are often limited by the preliminary step of DNA isolation. The obtainment of great amounts of high quality DNA from small quantities of tissue is often a laborious task.

DNA extraction methods should ideally be straightforward, quick, efficient, and reproducible while minimizing the potential for cross-contamination. It should also be suitable for extracting multiple samples and generate minimal risk for the operator. Safety, time and costs are also main considerations. DNA quality is a critical issue for most amplification-based analysis, since the DNA amplification is influenced by the presence of co-purifying inhibitors from matrix or extraction reagents, which can reduce subsequent PCR efficiency. DNA damage may also occur during the extraction procedure due to oxidation and enzymatic hydrolysis problems, associated with extraction buffers formulation [1] and excessive mechanical shearing [2].

The great majority of methods for DNA extraction were generated for human (especially blood samples) and for other mammalian or plant species [3]. The traditional methods for DNA extraction were time-consuming [4], and required the use of health hazard reagents and possible contaminants of the extracted genomic DNA. Phenol-chloroform extraction [5], salting out procedure [6], silica-guanidinium thiocyanate method [7,8], CTAB procedure [9] and Chelex-based extraction [10] are the most used protocols. Nowadays, commercial DNA extraction kits are available, employing a variety of solvents and/or specialized columns containing DNA-binding substances, procedures are shorter and easier to handle and does not require using toxic products, such as phenol [11].

Genetic research in marine invertebrates, such as molluscs, is scarce when compared to mammals. There are several difficulties in DNA extraction from molluscs that might contribute to this gap, such as its physiology and the type of tissue used (e.g., adductor muscle, foot muscle or gills). As an example, molluscs secrete mucopolysaccharides and polyphenolic proteins which copurify with DNA and interfere with enzymatic processing of nucleic acids [12]. Furthermore, molluscs do not often have large tissue pieces, consequently the availability of optimized protocols for DNA extraction is limited and alternative straightforward methods for genomic DNA extraction are crucial.

Throughout the years, an increasing number of specific protocols have been applied for DNA extraction from molluscs and related taxonomic groups. Essentially these protocols present modifications from other methods of DNA extraction in mammals or plants: the protocol presented by Winnepenninckx *et al.* [12] was modified from a plant DNA extraction protocol developed by Doyle [13]; the QIAGEN DNeasy Tissue Kit (Qiagen) and mi-Tissue Genomic DNA Isolation Kit (Metabion GmbH) have been applied to molluscs DNA extraction by Vasta *et al.* [14] and Popa *et al.* [15], respectively. More recently, some biotechnology companies have developed specific kits for bivalves DNA extraction (e.g., E.Z.N.A Mollusc DNA kit from Omega Bio-Tek), but in general they still need laborious handling and use of toxic reagents. The aim of our study was to develop an efficient and straightforward method for molluscs DNA extraction that would allow the obtainment of high molecular weight DNA with superior purity, especially using small quantities of tissue. The protocol presented here does not involve the use of toxic reagents (e.g., phenol or chloroform). Moreover, the resulting DNA is suitable for several molecular applications (e.g., PCR, cloning, sequencing), namely in large genetic population studies of mollucs. This method relies on the use of automatic system equipment (QuickGene-810) and the QuickGene DNA Tissue kit, both developed by Fujifilm Life Science.

## 2. Results and Discussion

The Automatic Nucleic Acid Isolation System (QuickGene 810, Fujifilm Life Science) associated with the modifications in QuickGene DNA Tissue kit were applied with great success to different and large numbers of mollusc species. Developed originally for mammalian and plant tissues, the important adaptations in critical steps that were performed in this study, such as lyses (use of the Pestle Pellet and 3–4 h of incubation) and elution times (increase of elution time in the automatic nucleic-acid isolation system QuickGene-810) ensured adequate digestion, elution and, consequently increased the DNA yield. The modifications introduced in the original protocol from QuickGene DNA Tissue allowed obtaining superior yields of high quality genomic DNA from small amounts of tissue, sufficiently pure and suitable for downstream molecular applications. The automation of DNA extraction has the advantage of standardized sample treatment and avoidance of error during routine sample handling and contamination due to intermediate processes [11]. The number of samples processed simultaneously (eight) make this ideal for large genetic population studies keeping the reproducibility and quality of the DNA isolated. This method did not generate hazardous waste (phenol and chloroform) and does not require any specific safety procedures since the user is not exposed to hazardous or noxious fumes, vapors or dusts.

### 2.1. Evaluation of the Genomic DNA Integrity by Agarose Gel Electrophoresis

**Figure 1:**
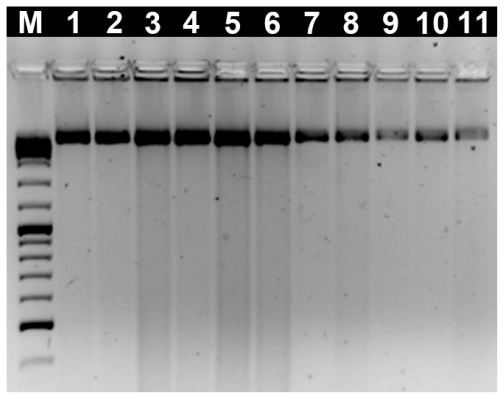
Electrophoresis of DNA extracts in 1.0% agarose gel. M: O’GeneRuler^TM^ DNA Ladder Mix (Fermentas); 1: *C. gallina*; 2: *V. aurea*; 3: *V. pullastra*; 4: *R. decussatus*; 5: *C. gigas*; 6: *O. stentina*; 7: *O. edulis*; 8: *O. chilensis*; 9: *A. ephippium*; 10: *C. edule*; 11: *H. trunculus*.

The integrity of all genomic DNA samples isolated from several individuals was analyzed by agarose gel electrophoresis. In Figure 1 it is possible to observe eleven examples of genomic DNA isolated from several species (*Crassostrea gigas*, *Ostrea stentina*, *Ostrea edulis*, *Ostrea chilensis*, *Chamelea gallina*, *Ruditapes decussatus*, *Venerupis pullastra*, *Venerupis aurea*, *Anomia ephippium*, *Cerastoderma edule* and *Hexaplex trunculus*) with almost no DNA fragmentation and a high molecular weight band.

### 2.2. Evaluation of the Genomic DNA Quantity and Quality by NanoDrop^®^ ND-1000 (NanoDrop Technologies)

Purity, concentration and yield of genomic DNA samples were estimated with NanoDrop^®^ ND-1000 (NanoDrop Technologies) with the purpose of evaluating parameters such as quality and quantity. The DNA purity and concentration was directly measured by NanoDrop^®^ ND-1000 system, while, the DNA yield was estimated, for each sample, comparing the quantity of genomic DNA obtained with the quantity of tissue used (cf. Material and Methods). The DNA isolation can be influenced by several factors like species, tissue preservation method and extraction procedure. In molluscs this procedure is known to be challenging due to the high amount of mucopolysaccharides and polyphenolic proteins present in these animals tissues.

From Table 1 it is possible to analyze the concentration, yield and purity of genomic DNA extracted from several species (*N* = 100). One of the important features of this protocol is the possibility of its application to a great variety of mollusc species. The average concentration of the total extracted genomic DNA of all samples was 271.8 ± 64.5 ng·μL^−1^ (mean ± SE), ranging from 200.7–370.3 ng·μL^−1^ (min-max). Typical DNA yield ranges from 1000–5000 ng·mg^−1^ in animal tissue [16]. For the mollusc analyzed in this study, the extraction method generated adequate DNA yield, ranging from 823.6–5053.8 ng·mg^−1^ (mean ± SE). Indeed, it is not easy to compare our results with the ones achieved by other methods and that are already published for mollusc species, as most of them use different types of tissues such as gills, rectum and mantle. In fact, these tissues are easier for DNA isolation but in the end the DNA could be contaminated with alien DNA as for example from parasites. Nevertheless, in recent works published [15,16] with protocols for bivalves DNA extraction, the total DNA measured ranges between 0.5–250 μg, indicating similar values of high molecular weight DNA using this protocol, a mean of 27.2 ± 6.5 μg (mean ± SE).

There are several types of contaminations that can be acquired during the DNA extraction protocols, depending on the origin of the biological sample [12,13]. Phenolics and other secondary compounds cause damage of the DNA and/or inhibit enzymatic reactions. The quality of the samples evaluated in terms of RNA/protein and chaotropic salt contamination was respectively, 1.88 ± 0.04 and 1.83 ± 0.12 (mean ± SE) (Table 1), being all samples analyzed around the optimal value, for both quality standards. These minor levels of salt, protein or RNA contamination prevent interference in downstream molecular biology procedures and so, it is very important to maintain its levels to a minimum. Riemann *et al*. [17] suggested that quantity and quality of the isolated DNAs were slightly higher with manual extraction than automatic extraction methods. In our experience we notice that the automatic extraction is more efficient regarding the quality of genomic DNA, which is very important to downstream molecular procedures.

One of the main objectives of the protocol presented was the maximization of this process for small amounts of tissue, since one of the problems of genetic studies in molluscs is the scarcity of tissue. All the data was clustered in five different groups of weights and the mean of concentration, DNA yield and purity was quantified (Table 2). As can be observed in Table 2, the data demonstrates that the total genomic DNA yield is optimal in intervals of [0–5] and [5–10] mg, respectively 6887.30 ± 613.72 and 3577.16 ± 490.42 ng·mg^−1^ (mean ± SE), meaning that with small quantities of tissue it was possible to obtain the highest yields of genomic DNA representing a prominent feature of this protocol. Moreover, the same company (*i.e.*, the QuickGene 610) developed a new automatic DNA extraction system that allows a ten-fold starting amount (compared with the QuickGene 810), so correspondingly bigger amounts of DNA may be achieved. However, this system permits only the simultaneous handling of six samples.

### 2.3. Evaluation of the Genomic DNA Isolated in Downstream Applications

The genomic DNA obtained with the presented methodology was of high quality regarding all standards employed. However, and as described by different authors, the quality and total DNA contents provided by NanoDrop do not accurately represents the quantity of DNA that is efficiently amplifiable by PCR [18,19]. In order to analyze the quality of amplifiable DNA, we PCR-amplified amplicons for the histone H3 gene in all samples. We also performed a random PCR that would ideally generate several DNA segments, like RAPDs, since this technique covers the entire genome.

**Figure 2:**
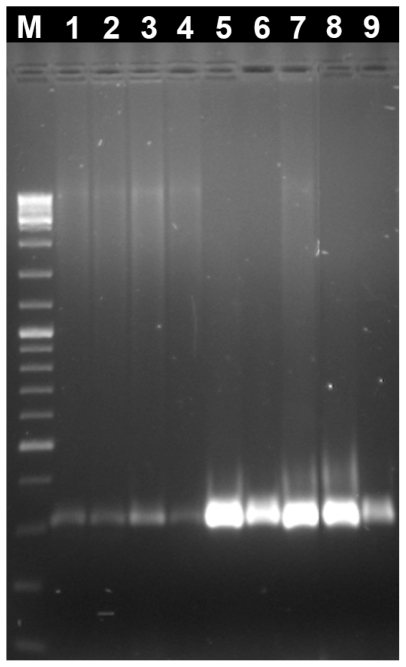
Agarose gel electrophoresis of PCR amplification of histone H3 gene in several molluscs species. M: O’GeneRuler™ DNA Ladder Mix (Fermentas); 1: *C. gallina*; 2: *V. aurea*; 3: *V. pullastra*; 4: *R. decussatus*; 5: *C. gigas*; 6: *O. stentina*; 7: *O. edulis*; 8: *O. chilensis*; 9: *H. trunculus*.

**Figure 3:**
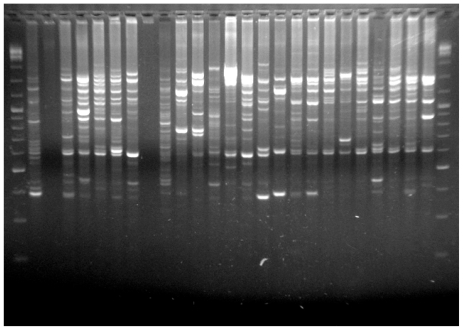
Agarose gel electrophoresis of PCR amplification of RAPDs in *R. decussatus*.

PCR conditions were optimized in the genomic DNA samples obtained by the present method and the histone H3 gene was amplified with success in all species (Figure 2). This technique was also successfully applied in DNA extraction in population genetics studies using RAPDs already published [20,21] (Figure 3). Moreover, we also sequenced, with great success, specific genome fractions, major and minor ribosomal genes isolated from genomic DNA prepared with the methodology described here, being elucidative of the quality of the genomic DNA obtained. These sequences are available in *GenBank* sequence database with the following access numbers: JN797504, JN797505, JN797506 and JN797507.

## 3. Experimental Section

### 3.1. Sample Collection

Several species of bivalves, *C. gallina* (*N* = 10), *V. aurea* (*N* = 10), *V. pullastra* (*N* = 10), *R. decussatus* (*N* = 10) (Bivalvia: Veneridae), *C. gigas* (*N* = 10), *O. stentina* (*N* = 10), *O. edulis* (*N* = 10), *O. chilensis* (*N* = 10) (Bivalvia: Ostreidae), *A. ephippium* (*N* = 5) (Bivalvia: Anomiidae), *C. edule* (*N* = 10) (Bivalvia: Cardiidae) and one gastropod, *H. trunculus* (*N* = 5) (Gastropoda: Muricidae) (*N* = number of individuals), were collected from Ria Formosa populations, Algarve, Portugal. After two days of depuration, the samples were processed and placed in 70% ethanol at −20 °C, until further use.

### 3.2. DNA Extraction Protocol

Fresh adductor muscle tissue from different bivalves was used for DNA extraction, while for gastropod *H. trunculus* egg capsules were used. To extract the genomic DNA from all the animals we used the Automatic Nucleic Acid Isolation System (QuickGene 810, Fujifilm Life Science). This system uses a porous ultra thin membrane and an automatically pressurizing unit that promotes binding, washing and elution steps at low pressure. In order to apply this system to the isolation of genomic DNA from molluscs, several modifications were carried out: (a) the use of the “Pestle Pellet” and the adaption of 3–4 h of incubation ensured a more efficient digestion; (b) the increase of elution time in the automatic nucleic-acid isolation system QuickGene-810 was more efficient for the elution of genomic DNA, and consequently for the increasing of the DNA yield. Tissue samples preserved in 70% ethanol, were washed with 1× PBS and distilled water for 10 min each. A section of tissue (about 5–30 mg of tissue) was cut in small pieces followed by the addition of 180 μL of MDT (tissue lysis buffer) and 20 μL of EDT (buffer with Proteinase K) in a 2 mL eppendorf. The samples were homogenised with the aid of a “Pellet Pestle”, vortexed briefly and incubated at 55 °C between 3 and 4 h. The eppendorfs were removed from incubation and at this point if any debris are present at the lyses, is recommended to remove it by centrifugation (10,000 g, 3 min). The supernatant was carefully transferred to a new 2 mL eppendorf. A volume of 180 μL of LDT (buffer solution) was added and mixed thoroughly. This procedure must be performed in a vortex during 15 s and followed by a quick spin down. The solution was then incubated at 70 °C during 10 min and occasionally mixed with a vortex. At the end of the incubation step, a quick spin down was performed. An ethanol volume of 240 μL of 100% (v/v) was added and mixed very well. The lysate was then transferred to a cartridge of the automatic nucleic-acid isolation system QuickGene-810 and the “DNA tissue mode” was selected with a major modification in the elution time to maximum. A standardized final volume of 100 μL was used and the samples of genomic DNA were ready to be used immediately or stored at −20 °C for several months.

### 3.3. DNA Analysis

NanoDrop ND1000 Spectrophotometer (NanoDrop Technologies, Inc.) was used to measure the absorbance. The values of absorbance (A) allowed estimating the purity, concentration and yield of the genomic DNA samples. Pure DNA exhibited an A_260_/A_280_ ratio (RNA/protein contamination) and an A_260_/A_230_ ratio (chaotropic salt contamination) in the range of 1.8–2.0. To compare the efficiency of DNA extraction on various tissue weights, the DNA yield (DNA_ng_/Tissue weight_mg_) was estimated.

To assess the DNA quality, several standard molecular laboratory procedures were performed: a PCR for amplification of the histone H3 gene and RAPDs according to Zhang *et al.* 2007 [22] and Pereira *et al.* 2010 [21], respectively. The integrity of the genomic DNA samples extracted as well as the PCR amplification samples were analyzed by electrophoresis on a 2% agarose gel with O’GeneRuler^TM^ DNA Ladder Mix (Fermentas, Glen Burnie, MD, USA). After electrophoresis run at 75 volts, for 1 h, the DNA bands were observed under UV light and the images were saved in a gel analyser (UVIDOC).

## 4. Conclusions

Reliability, feasibility and reproducibility of molecular genetics studies depend on high molecular weight and high quality genomic DNA with low levels of fragmentation and DNA efficiently amplifiable by PCR. In this work, we obtained genomic DNA with high purity and yield, with low salt contamination, from different small amounts of tissues of molluscs. This protocol of genomic DNA extraction has a great potential to be applied in different molecular studies, especially in genetic populations studies which require a large number of DNA extractions, sometimes with low quantity of tissue, in reduced time, with high quality of genomic DNA.

## Figures and Tables

**Table 1:** Concentration and purity of genomic DNA extracted from several samples of different species.

Family	Species Names	*N*	DNA Concentration (mean ± SE) (ng·μL^−1^)	Amount Tissue (mean) (mg)	DNA Yield (mean ± SE) (ng·mg^−1^)	Evaluation RNA/Protein Contamination (A_260/280_) (mean ± SE)	Evaluation of Chaotropic Salt Contamination (A_260/230_) (mean ± SE)	PCR
Ostreidae	*C.gigas*	10	200.7 ± 30.2	16.80	2386.3 ± 778.0	1.91 ± 0.03	1.99 ± 0.07	+
	*O.stentina*	10	370.3 ± 118.6	16.80	3687.5 ± 1111.6	1.92 ± 0.03	1.94 ± 0.05	+
	*O. edulis*	10	331.6 ± 44.2	16.80	3426.1 ± 1136.4	1.85 ± 0.04	1.72 ± 0.10	+
	*O. chilensis*	10	256.4 ± 68.1	16.80	2795.5 ± 1001.0	1.89 ± 0.04	1.94 ± 0.11	+
Veneridae	*C. gallina*	10	279.7 ± 60.8	16.80	2739.7 ± 896.9	1.80 ± 0.04	1.72 ± 0.09	+
	*R. decussatus*	10	246.1 ± 44.6	16.80	2547.3 ± 832.9	1.89 ± 0.04	1.71 ± 0.10	+
	*V. aurea*	10	241.8 ± 40.5	16.80	2634.1 ± 923.4	1.91 ± 0.03	1.74 ± 0.09	+
	*V. pullastra*	10	254.7 ± 36.7	16.80	2688.4 ± 890.7	1.93 ± 0.05	1.78 ± 0.13	+
Anomiidae	*A. ephippium*	5	327.8 ± 111.5	11.60	5053.8 ± 2566.5	1.85 ± 0.03	1.83 ± 0.17	+
Cardiidae	*C. edule*	10	244.7 ± 52.3	16.80	2823.1 ± 1155.5	1.85 ± 0.03	1.86 ± 0.12	+
Muricidae	*H. trunculus*	5	247.1 ± 89.7	30.00	823.6 ± 299.0	1.90 ± 0.04	1.90 ± 0.21	+
	Total	100	271.8 ± 64.5		2695.8 ± 884.5	1.88 ± 0.04	1.83 ± 0.12	

**Table 2:** Concentration and purity of genomic DNA extracted from several samples clustered by weights.

Weight Class (mg)	*N*	DNA Concentration (mean ± SE) (ng·μL^−1^)	DNA Yield (mean ± SE) (ng·mg^−1^)	Evaluation RNA/Protein Contamination (A_260/280_) (mean ± SE)	Evaluation of Chaotropic Salt Contamination (A_260/230_) (mean ± SE)
[0–5]	25	344.8 ± 30.7	6887.3 ± 613.0	1.88 ± 0.02	1.82 ± 0.07
[5–10]	25	328.3 ± 50.4	3577.2 ± 490.4	1.90 ± 0.03	1.82 ± 0.05
[10–15]	25	259.0 ± 62.8	1871.2 ± 416.92	1.90 ± 0.03	1.75 ± 0.08
[15–20]	25	177.0 ± 21.1	935.5 ± 113.15	1.88 ± 0.03	1.81 ± 0.09
[20–25]	25	245.3 ± 38.0	771.3 ± 159.1	1.84 ± 0.04	1.91 ± 0.05
Total	100	271.8 ± 40.6	2808.8 ± 358.6	1.88 ± 0.04	1.82 ± 0.12
